# No Benefits in Using Magnetically Controlled Growing Rod as Temporary Internal Distraction Device in Staged Surgical Procedure for Management of Severe and Neglected Scoliosis in Adolescents

**DOI:** 10.3390/jcm12165352

**Published:** 2023-08-17

**Authors:** Pawel Grabala, Kelly Chamberlin, Michal Grabala, Michael A. Galgano, Ilkka J. Helenius

**Affiliations:** 1 Department of Pediatric Orthopedic Surgery and Traumatology, University Children’s Hospital, Waszyngtona 17, 15-274 Bialystok, Poland; 2 Paley European Institute, Al. Rzeczypospolitej 1, 02-972 Warsaw, Poland; 3 Department of Neurosurgery, University of North Carolina, Chapel Hill, NC 27516, USA; kelly.chamberlin@unchealth.unc.edu (K.C.); mgalgano@email.unc.edu (M.A.G.); 4 2nd Clinical Department of General and Gastroenterogical Surgery, Medical University of Bialystok, ul. M. Skłodowskiej-Curie 24a, 15-276 Bialystok, Poland; michal@grabala.pl; 5 Department of Orthopedics and Traumatology, Helsinki University Hospital, 00260 Helsinki, Finland; ilkka.helenius@helsinki.fi

**Keywords:** severe scoliosis, Halo, HGT, MCGR, scoliosis, spinal deformity, temporary internal distraction, neglected scoliosis

## Abstract

Background: Severe spinal curvatures (SSCs) in children and adolescents have long been treated with preoperative Halo traction, in its various variations. There are also several radical techniques available for the management of neglected SSCs, such as osteotomies; however, these can be risky. Comparing the treatment outcomes when using preoperative Halo Gravity Traction (HGT) against the use of a Magnetically Controlled Growing Rod (MCGR) as a temporary internal distraction (TID) device, we evaluated the differences in surgical and radiological outcomes. Methods: We conducted a retrospective study of 30 patients with SSCs, treated with HGT followed by posterior spinal fusion (PSF; Group 1, *n* = 18) or treated using a temporary MCGR as a TID followed by PSF (Group 2, *n* = 12). All patients underwent surgical treatment between 2016 and 2022. The inclusion criteria were SSC > 90°, flexibility < 30%, and the use of preoperative HGT followed by PSF or the two-stage surgical procedure with initial TID rod placement (Stage 1) followed by PSF (Stage 2). The evaluated parameters were as follows: rib hump, trunk height, and radiographic outcomes. All parameters were collected preoperatively, after the initial surgery, after final correction and fusion, and during the final follow-up. Results: In Group 1, we evaluated 18 patients with a mean age of 15.5 years; in Group 2, we evaluated 12 patients with a mean age of 14.2 years. The interval between the staged procedures averaged 32.7 days. The mean preoperative main curves (MC) were 118° and 112° in Group 1 and Group 2, respectively. After definitive surgery, the MC was corrected to 42° and 44° in G1 and G2, respectively. The mean percentage correction of the MC was similar in both groups (65% vs. 61% in G1 and G2, respectively). The mean preoperative thoracic kyphosis was 92.5° in G1 and 98° in G2, corrected to 43.8° in G1 and 38.8° in G2. Trunk height increased by 9 cm on average. Conclusions: There are no benefits in using a MCGR as a temporary internal distraction device in the management of neglected scoliosis in adolescents. Surgical treatment of severe scoliosis may be safe, with a reduced risk of potential complications, when using preoperative HGT. A specific intraoperative complication when using a MCGR as a temporary internal distraction device was a 50% risk of transient neuromonitoring changes, due to significant force applied to the spine and radical distraction of the spine. We achieved similar clinical, radiographic, and pulmonary function outcomes for both techniques. The use of HGT causes less blood loss with a shorter overall time under anesthesia. Partial correction significantly aids the subsequent operation by facilitating a gradual reduction in the curvature, thereby reducing the difficulty of surgical treatment and the risk of neurological deficits.

## 1. Introduction

The treatment of severe (and often neglected) spinal deformities in children and adolescents can be a real challenge for the spine surgeon and the entire team, as it is associated with a higher risk of intra- and postoperative complications [1,2,3,4]. Severe spinal curvatures have long been treated with preoperative Halo spinal traction, in its various variations, such as Halo femoral traction (HFT), Halo pelvic traction (HPT), or Halo gravity traction (HGT) [5,6,7,8,9,10]. With the introduction of modern magnetically controlled growing rods in spinal surgery, they have also been adopted and described in the literature as temporary internal distraction devices for the treatment of severe scoliosis [11,12].

As we did not find any studies in the existing reports comparing the outcomes of treatment of severe spinal deformities in children and adolescents using HGT compared to the use of a Magnetically Controlled Growing Rod (MCGR) as a temporary internal traction device, we decided to evaluate two groups of patients treated by our team using these two methods to better delineate, identify, and isolate the differences between the two techniques. We hypothesize that staged surgery with MCGR temporary internal distraction (TID) would provide similar outcomes for severe scoliosis as compared with preoperative HGT.

## 2. Materials and Methods

### 2.1. Setting, Patients, and Measures

This retrospective study was approved by the ethics committee of the district hospital (approval No. APK.002.80.2020) and included patients treated surgically for severe and neglected scoliosis, who had not been previously treated surgically. All children had previously undergone conservative treatment. All patients in this study and their parents gave written informed consent to the publication of our study results. A total of 30 patients from the pediatric population were selected as the study group. Among all the patients included in the study, Group 1 (*n* = 18) used a preoperative Halo gravity traction, and Group 2 (*n* = 12) used a temporary internal distraction system with a MCGR. Inclusion criteria were severe spinal deformities (major Cobb curve > 90°) with flexibility < 30%, and use of preoperative HGT followed by PSF or two-stage surgery with initial TID (Stage 1) followed by PSF (Stage 2). All patients underwent surgical treatment in the years 2016–2022 at the Children’s Hospital. All procedures were performed by an experienced pediatric orthopedic surgeon.

Among the analyzed patients, we took into account the basic parameters of the chest, we measured spinal deformity-specific parameters such as the Cobb angle of the proximal thoracic, main thoracic, and lumbar curvatures, as well as measurements in the sagittal plane—thoracic kyphosis (T5–T12) and lumbar lordosis (T12–S1). The flexibility of the spine was measured using bending films. The rib hump measurement, trunk height, and vertebral apical translation (AVR) were also noted and analyzed. The collected parameters were analyzed before surgery, after the first surgery, after the final correction and fusion, and in the final follow-up period. Spinal deformities of idiopathic etiology were classified according to Lenke’s classification [13]. Radiographic measurements were performed by an independent observer. Preoperative MRI of the entire spine revealed or excluded other pathologies of the spinal cord. All patients underwent intraoperative spinal cord monitoring, including somatosensory evoked potentials (SSEP) and transcranial motor evoked potentials (MEP) [14].

### 2.2. Statistical Analysis

Statistical analysis software (version 10.0; StatSoft Inc., Tulsa, OK, USA) was utilized for all analyses. Parametric (one-way analysis of variance) and nonparametric (Mann–Whitney U) analyses were performed to compare the results between groups. The data were also analyzed using the ANOVA test and the Tukey–Kramer method. For analysis and presentation of the data, we used the standard deviation (SD) of the mean, 95% confidence interval (CI), medians with lower and upper quartiles, or frequency. The assumption of normal distribution was tested together using the Shapiro–Wilk test, and the Mann–Whitney U test and the Kruskal–Wallis analysis in the variance rank test were conducted for comparisons between groups. Pearson’s correlation coefficients were calculated to study the relationship between two numerical variables. Changes between the two time points were compared using McNemar’s tests. A *p*-value < 0.05 was considered to indicate statistical significance.

### 2.3. Surgical Technique

**Figure 1:**
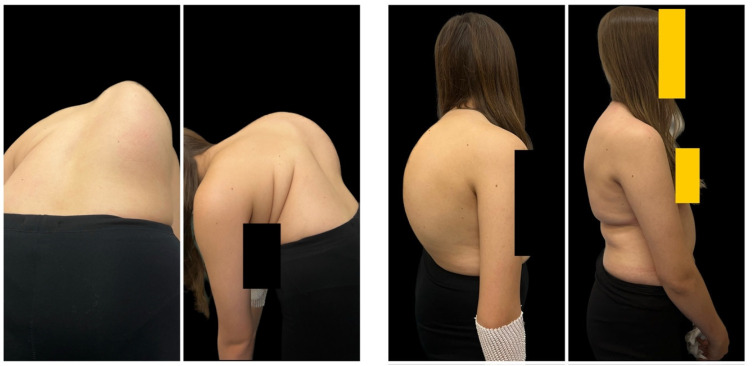

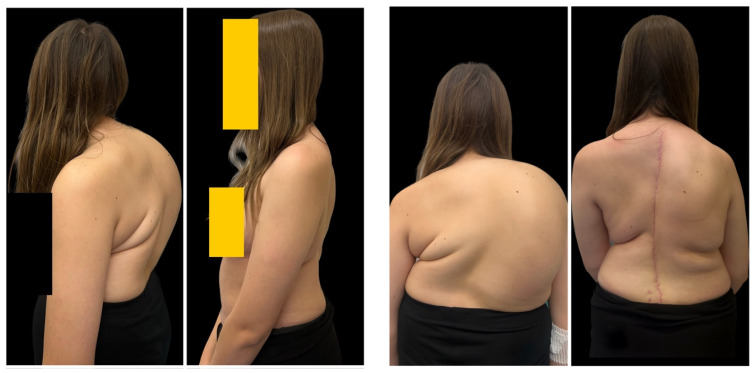
A 14-year-old girl with severe adolescent idiopathic scoliosis treated with preoperative Halo gravity traction followed by multilevel Ponte osteotomies and final correction with double Co-chr 6.0 rods. Pre- and postoperative clinical photographs during the observation period.

**Figure 2:**
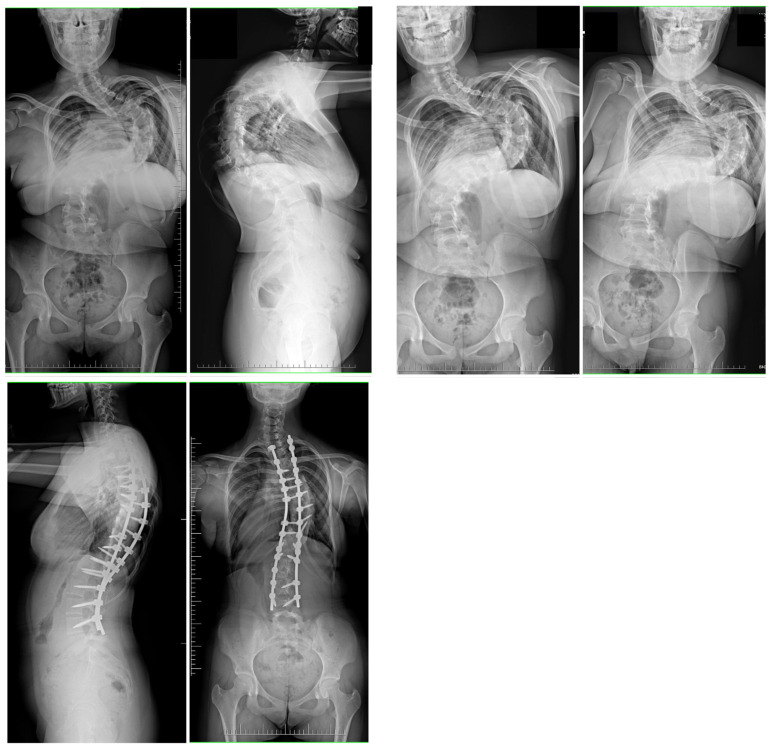
A 14-year-old girl with severe adolescent idiopathic scoliosis treated with preoperative Halo gravity traction followed by multilevel Ponte osteotomies and final correction with double Co-chr 6.0 rods. Pre- and postoperative radiographs during observation period.

For the patients in Group 1, the initial Halo ring was placed under general anesthesia, and traction was started with a weight of 2 kg and gradually increased at a rate of 1 to 2 kg per 1–3 days if the patients showed sufficient tolerance (active traction) and 2–3 kg for the night as supporting traction, as described in the literature [15,16]. The duration of HGT treatment was determined by the patient’s adaptation and tolerance to HGT and by increasing the traction weight to a maximum of 50% of the patient’s body weight. After finishing the HGT course, we performed correction of the spinal deformity through a posterior approach using segmental screw instrumentation. After dissecting the soft tissues and gaining access to the spine, facetectomies were performed at all levels, and pedicle screws were inserted into individual vertebrae. An osteotomy of the posterior column of the spine (Ponte) was performed, usually at all levels [17,18]. The correction was performed by synchronous two-rod derotation with neuromonitoring after appropriate profiling of the rods. Other maneuvers, such as compression, distraction, translation, and in situ bending of the rods, were also performed in the correction [19]. The wound was then closed by layered suturing. Postoperative immobilization was not used. Pre- and postoperative clinical images are shown in Figure 1, and pre- and postoperative radiographs are shown in Figure 2.

The surgical treatment of patients from Group 2 was carried out in two stages. The first surgical procedure consisted of a wide standard access to the spine from the posterior approach, exposing the spine at the planned stabilization levels. A facetectomy was performed at all levels of the spine on both left and right sides, excluding the upper instrumented vertebrae (UIV). A typical Ponte osteotomy, as described in the literature [17,18], was performed at all levels and segmental screws were implanted at all levels—at least on one side. On the concave side of the curvature, a properly bent MCGR was placed, achieving partial correction. The procedure was performed under the control of neuromonitoring. The pedicle screws on the two levels—upper and lower on the convex side—were connected with a temporary short stabilizing rod, creating two rigid blocks and strengthening the structure for future spinal distraction. The patient was discharged home on the fifth day; then, once a week for the next 6 weeks, gradual spinal distractions were performed until the maximum moment of MCGR distraction was reached. Each distraction was performed with the maximum possible torque, or until the “clunking phenomenon” was noticed [20]. The forces acting during spinal distraction were very high. One distraction control image was taken after 6 weeks of gradual lengthening of the magnetic rod.

**Figure 3:**
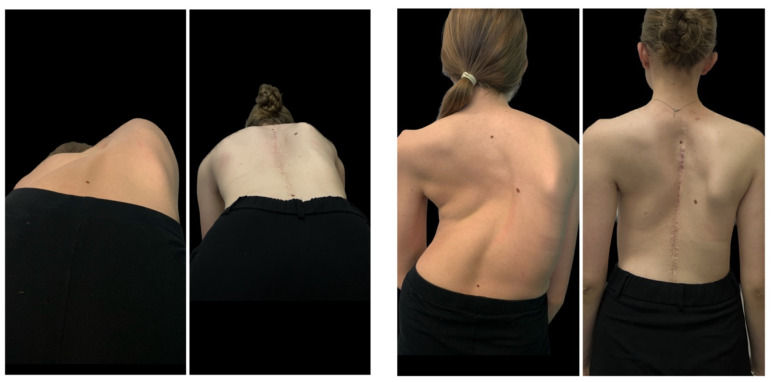

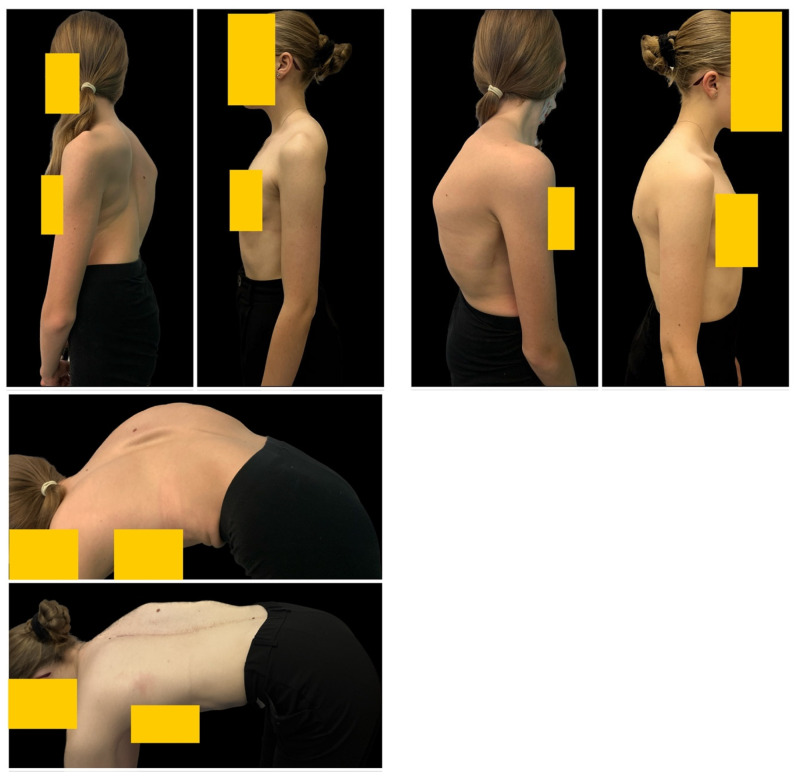
A 16-year-old girl with severe adolescent idiopathic scoliosis treated with staged surgery using MCGR as temporary internal distraction followed by final correction with double Co-chr 6.0 rods. Pre- and postoperative clinical photographs during observation period.

**Figure 4:**
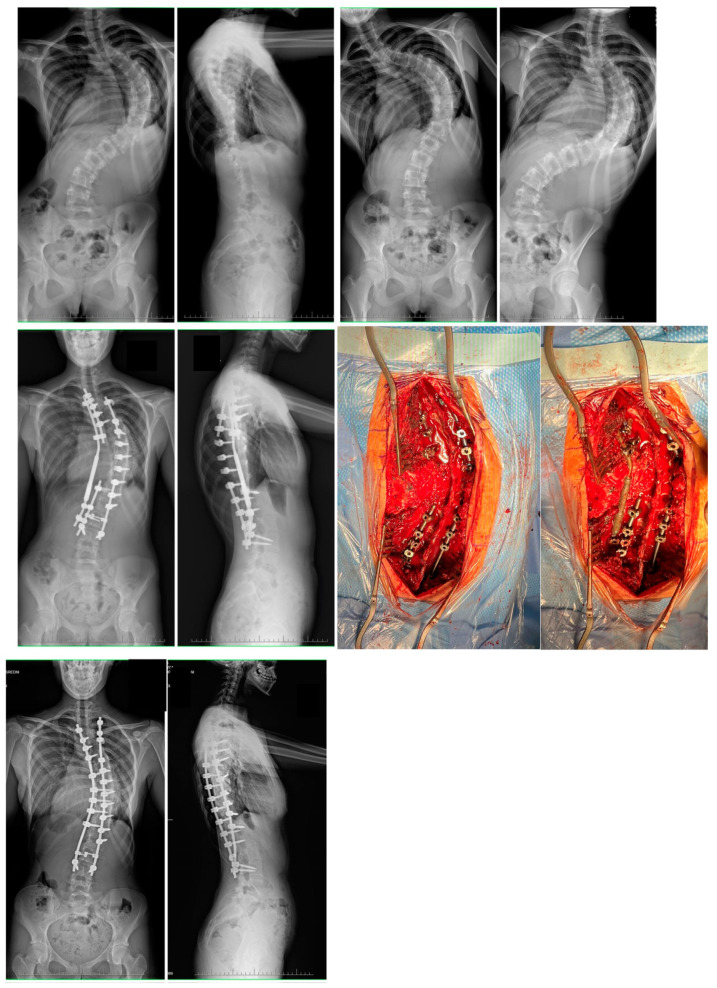
A 16-year-old girl with severe adolescent idiopathic scoliosis treated with staged surgery using MCGR as temporary internal distraction followed by final correction with double Co-chr 6.0 rods. Pre- and postoperative radiographs during observation period and intraoperative pictures shown using MCGR as a temporary internal distraction device.

After further distractions of the MCGR were exhausted, a final correction of the spinal deformity was performed (including removal of the MCGR), and the final correction was achieved by a combination of rod distraction/compression, apical translation, and segmental derotation. During the second treatment, the resulting distraction length (mm) of the rod was measured with a ruler (Figure 3 and Figure 4).

## 3. Results

### 3.1. Clinical Characteristics and Radiographic Outcomes

We analyzed and compared a total of 25 girls and 5 boys with severe spinal deformities with a mean follow-up of 2.9 years. A total of 18 patients (17 girls, 1 boy) with a mean (SD) age of 15.5 (6.5) years were placed in Group 1, while 12 patients (8 girls, 4 boys) with a mean (SD) age of 14.2 (6.8) years were included in group 2 (Table 1). The interval between successive treatments was 32.7 days (21–42 days) on average. The mean (SD) preoperative MC was 118° (8.4) and 112° (8.9) in groups 1 and 2, respectively (*p* = 0.672). The mean preoperative flexibility in preoperative bending films ranged from 18% (7.2) in G1 to 21% (15.5) in G2 (NS).

At final surgery, MC was corrected to 42° (12.6) and 44° (9.4) in G1 and G2, respectively (*p* = 0.821). The mean percentage correction of MC was similar in both groups (65% vs. 61% in G1 and G2, respectively), with no statistically significant difference between the groups (NS). No significant progression of MC was observed during follow-up in either group (Table 2). The mean preoperative thoracic kyphosis was 92.5° (9.8) in G1 and 98° (8.8) in G2 (*p* = 0.942). It was corrected to 44° (14.9) in G1 and 39° (8.2) in G2 (*p* = 0.611). The mean preoperative lumbar lordosis was −62° (24.8) in G1 and −49° (9.8) in G2 (*p* = 0.41), which was corrected to −42° (11.8) in G1 and −39° (12.8) in G2 (*p* = 0.251). The mean preoperative apical shift improved from 72 mm (22.4) to 33 mm (16.9) at the last follow-up visit at G1, and from 68 mm (18.8) to 34 mm (22.4) at the last follow-up visit in G2 (*p* < 0.001). There was significant correction rate received for both groups between preoperative parameters and at the follow-up (*p* < 0.001). Representative cases are shown in Figure 1 and Figure 2 (HGT and PSF), and Figure 3 and Figure 4 (TID and PSF). Detailed results of all radiographic measures are summarized in Table 2.

### 3.2. Complications

We found statistical differences between the groups in neuromonitoring changes during correction. Intraoperative neuromonitoring changes were noted in 50% of patients in Group 2 and 16.6% of patients in Group 1 (*p* < 0.001). The NM changes were related to the distraction of the deformed spine. After decreasing spinal distraction, NM responses returned to normal. None of the patients presented a new postoperative neurological deficit in either group, but pneumonia was observed in 11% vs. 25% of the groups, respectively (*p* < 0.001; Table 3). In Group 1, we noted 27.8% superficial infections around the pins; the patients were treated with oral antibiotics. In Group 2, we noted 25% superior mesentery artery syndrome (SMAS): two patients after initial insertion of the MCGR and correction during rod placement, and one patient after definitive surgery. None of the patients required any special treatment other than a special diet. No additional complications were noted during the final follow-up.

## 4. Discussion

This retrospective analysis demonstrated similar clinical outcomes, radiographic outcomes, and pulmonary function using either a preoperative Halo gravity traction period or staged surgery with temporary internal distraction using a MCGR for severe scoliosis. A specific intraoperative complication when using a MCGR as a temporary internal distraction device was a 50% risk of transient neuromonitoring changes due to the significant force applied to the spine, radical distraction of the spine, and staged surgery. The use of HGT causes less blood loss and shorter overall time under anesthesia.

Various protocols for preoperative traction exist, and the course of treatment with traction can also differ [5,6,7,8,9,10,15]. Many studies have confirmed the legitimacy and need for the use of HGT in the treatment of severe deformities [21]. In the available literature, Liu et al. [6] reported the results of treating severe spinal deformity with preoperative HGT and compared subsequent surgical treatment in adolescents and adult patients with severe scoliosis. The analyzed patients presented a significant improvement in measurements of the main curve of scoliosis and kyphosis. Postoperative neurological complications occurred in 18.2–27.6% of treated patients. In the adolescent group, the mean correction of the principal curvature improved from 139° before traction to 59° after surgical correction and anastomosis, and the angle of thoracic kyphosis was corrected from 130° before traction to 48° after surgery [6]. Other comparative studies [15] obtained similar results in terms of improvements in the Cobb angles. The coronal and sagittal major curves after treatment were reduced by 54.7% and 44.2%, respectively, during final surgery. After definitive surgery with PSF, the correction of the main curvature was 49% [7]. These results are similar to the data obtained in our study, as we noted fewer neuromonitoring changes in Group 1 than in Group 2. LaMont et al. [8] studied 107 patients undergoing HGT and PSF. Patients were treated with HGT for an average of 82.1 days, and the mean maximum percentage body weight in the extract was 49.5%. The mean greater coronal Cobb angle before HGT was 92.6°, while, after surgical intervention it improved to 47° [8]. In another study [22], the mean degree of the Cobb angle improved from 99.9° preoperatively to 49.5° postoperatively. The angle of kyphosis was corrected from 56° to 38° [22]. Some authors reported that preoperative kyphosis was 91° on neutral radiographs, and the degree of post-operative kyphosis was 70°, while major scoliosis before HGT was 106°, which, after definitive surgery, was corrected to 98° [5]. In a large meta-analysis [21] comparing patients who underwent PSF with pre-traction HGT values, a significant reduction in Cobb angle, a decrease in thoracic kyphosis, improvements in spine height, coronal balance, and pulmonary function, and an increase in nutritional status (BMI) were noted with preoperative Halo gravity traction in patients with severe spinal deformity.

The use of a gradual spinal lengthening system with MCGR in severe AIS can be considered an alternative technique for the treatment of severe scoliosis with a relatively high risk of transient neuromonitoring changes, allowing for gradual correction of the curvature prior to final posterior surgery with fusion, compared to an increasing risk of the neurological complications associated with more aggressive one-stage surgeries without preoperative HGT. A MCGR as a TID can eliminate long-term hospital treatment and apply a greater traction force to the spine taking benefits of its viscoelastic properties after applying the posterior release (PCO), which provides powerful traction mechanisms to the deformed spine [11,12,23,24]. Related disadvantages that not everyone may accept include phased treatment, two operations under anesthesia, a stay in the operating block, and a higher risk of decreases in spinal cord neuromonitoring potentials during two treatment courses. In the literature, Koller et al. evaluated this treatment technique [11] in seven patients with a major curve >100° treated with a temporary MCGR. The preoperative mean main curve was 118°. Patients underwent staged treatment for severe scoliosis. No major complications or neurological deficits were noted. This type of staged surgery achieved a correction of the postoperative main curve to 43.8° on average for a main curve correction of 67%. Spinal height T1-LIV increased by more than 10 cm [11]. In the study by Di Silvestre et al., the main scoliosis curves from an average pre-operative Cobb angle of 98.2° bent down to 38.3° after definitive fusion, and at last FU, the overall correction was 58.7%, with an average correction loss of 2.1° [12].

Both patients from Group 1 and Group 2 achieved satisfactory results, with an acceptable risk of minor complications. Both surgical techniques give good treatment results, and the final effect is comparable with no statistical differences in surgical and radiological parameters, but aggressive surgical procedures may cause more intra- and postoperative complications. Preoperative HGT allows for partial, less invasive, and safer correction of large and rigid curvatures, often with compensatory curvatures, thanks to which the final correction and fixation with transpedicular screws can be performed on a less sharp and rigid curvature [5,7,8,15,25]. There are patients who—for various reasons—cannot tolerate a long stay in the hospital and/or treatment with the traction (cervical instability) [5,7,21,26]. In such cases, the described technique can be a valuable alternative to HGT. First, treatment with the use of a MCGR as a temporary internal distraction device entails the need for two surgeries with a wide opening of the spine. This is associated with greater bleeding, a doubled time of stay in the operating room, and twice the postoperative care in the hospital. It is well known from the literature that each subsequent extensive surgical intervention in the spine increases the risk of infections and other complications [27]. Comparing perioperative complications in the two groups, a statistically significant higher number of complications occurred in patients in Group 2, which ultimately did not affect the result at the final follow-up. However, changes in spinal cord neuromonitoring were more frequent in Group 2 patients than those treated with preoperative HGT, which was related to the mechanism of action of the technique. When using the internal traction technique with a MCGR, a radical release of the spine occurs in the first stage, followed by multilevel PCO and then strong and radical traction on the spine, as far as neuromonitoring allows us. In the case of HGT, the spine is released after the HGT has been activated and the spinal cord and its blood supply have adapted to the new conditions.

### Limitations

Our study had some limitations due to its retrospective nature and small number of patients. It should be borne in mind that it is difficult to obtain a large number of patients with severe scoliosis of more than 90 degrees. Despite the occurrence of minor complications—which were fully acceptable to patients and their families—the results of treatment and our study are promising and justify further study into technical nuances, defining more specific and detailed indications.

The strength of this study is the careful analysis of the operated patients using two different surgical techniques with multiple outcome parameters including clinical and radiographic results. All patients participating in the study were treated, considered for qualification for surgical treatment, and operated on by the same experienced surgeons using the same surgical techniques. All patients were under constant observation after surgery for an average of 3 years.

## 5. Conclusions

There are no benefits in using a MCGR as a temporary internal distraction device in the management of neglected scoliosis in adolescents. Surgical treatment of severe scoliosis may be safe, with a reduced risk of potential complications, when using preoperative HGT. A specific intraoperative complication when using a MCGR as a temporary internal distraction device was a 50% risk of transient neuromonitoring changes, due to significant force applied to the spine and radical distraction of the spine. We achieved similar clinical and radiographic outcomes for both techniques. The use of HGT causes less blood loss with a shorter overall time under anesthesia. Partial correction significantly aids the subsequent operation by facilitating a gradual reduction in the curvature, thereby reducing the difficulty of surgical treatment and the risk of neurological deficits.

## Figures and Tables

**Table 1:** Patient demographics.

	Group 1 (*n* = 18)	Group 2 (*n* = 12)	*p*
Sex Male Female	1 17	4 8	
Mean age at surgery, years (SD), range	15.5 (6.5) 12.8–17	14.2 (6.8) 10–18	
Mean (SD) follow-up, years	3.28 (1.6)	2.6 (0.8)	
Mean BMI at surgery (SD), range	22.1 (6.7) 13–36	22.6 (5.5) 14–38	
Mean amount of segment involvement fusion (SD), range	13.2 (2.8) 12–15	12 (2.5) 11–15	
Percentage (*n*) of patients fused below L3	64%	58%	NS
Etiology of scoliosis I—idiopathic C—congenital N—neuromuscular S—syndromic	I—13 C—2 N—1 S—2	I—7 C—2 N—1 S—2	
Mean duration/stay at hospital, days (SD), range	42.5 (8.8) 28–62	First stage 6 (2.5) 4–9 Second stage 5 (1.8) 4–11	
Mean duration of surgery, min (SD), range	HGT 22 (12.5) 12–48 Final surgery 352 (72.8) 228–466	First stage 322.5 (84.8) 265–372 Second stage 281 (62.8) 211–321	
Mean blood loss at surgery, mL (SD), range	542 (280) 290–1280	First stage 611 (262.6) 310–1520 Second stage 328 (255) 220–820	
Mean total HGT duration, days (SD), range	35 (7.2) 28–52	NA	
Mean total MCGR distractions, cm (SD), range	NA	2.5 (1.2) 2.2–3.2	

**Table 2:** Radiological parameters before surgical treatment (pre- and postoperative) and at final follow-up.

	Group 1 (*n* = 18)	Group 2 (*n* = 12)	*p*-Value (G1 vs. G2)
Mean (SD) preoperative Cobb, °	118 (8.4)	112 (8.9)	0.672
Mean (SD) Cobb after initial distraction (Halo, MCGR), °	72.2 (22.6)	54 (8.2)	<0.001
Mean (SD) Cobb after definitive fusion, °	42 (12.6)	43.8 (9.4)	0.821
Mean (SD) Cobb at final follow-up, °	43.8 (9.2)	44.5 (7.2)	0.922
*p*-Value (preop vs. final follow-up)	<0.001	<0.001	
Mean (SD) major preoperative thoracic kyphosis, °	92.5 (9.8)	98 (8.8)	0.942
Mean (SD) major thoracic kyphosis after initial distraction (Halo, MCGR), °	72.5 (22.8)	55 (12.8)	<0.001
Mean (SD) major thoracic kyphosis after definitive fusion, °	43.8 (14.9)	38.8 (8.2)	0.611
Mean (SD) major thoracic kyphosis at final follow-up, °	42 (17.8)	36.3 (6.4)	0.128
*p*-Value (preop vs. final follow-up)	<0.001	<0.001	
Mean (SD) preoperative lumbar lordosis T12-S1, °	−62.1 (24.8)	−49 (9.8)	0.41
Mean (SD) lumbar lordosis T12–S1 after initial distraction (Halo, MCGR), °	−59.5 (26.2)	−46 (10.2)	<0.001
Mean (SD) lumbar lordosis T12–S1 after definitive fusion, °	−42 (11.8)	−38.8 (12.8)	0.251
Mean (SD) lumbar lordosis T12–S1 at final follow-up, deg°	−46.8 (12.8)	−42.8 (10.1)	0.322
*p*-Value (preop vs. final follow-up)	<0.001	0.287	
Mean (SD) preoperative apical vertebral translation, mm	72 (22.4)	68.2 (18.8)	0.192
Mean (SD) apical vertebral translation after initial distraction (Halo, MCGR), mm	55.8 (22.6)	58.2 (19.8)	0.627
Mean (SD) apical vertebral translation after definitive fusion, mm	31.6 (18.2)	32.2 (18.8)	0.931
Mean (SD) apical vertebral translation at final follow-up, mm	33 (16.9)	33.8 (22.4)	0.992
*p*-Value (preop vs. final follow-up)	<0.001	<0.001	

**Table 3:** Rate of complications following posterior final fusion.

Complication Rates Following Posterior Final Fusion	Group 1 (*n* = 18)	Group 2 (*n* = 12)	*p*
Intraoperative neuromonitoring changes	3 (16.6%)	6 (50%)	<0.001
Superficial wound infection	1 (5.5%)	2 (16.6%)	NS
Pneumonia	2 (11%)	3 (25%)	<0.001
Paresthesia from the lateral cutaneous nerve of the lower limb	3 (16.6%)	2 (16.6%)	NS
Pin infections	5 (27.8%)	NA	NS
Deep infection	0	1 (8%)	NS
SMAS	0	3 (25%)	NS
Total	14 (77%)	17 (141%)	<0.001

## Data Availability

Not applicable.

## References

[1] Amanullah A., Piazza M., Qutteineh B., Samdani A.F., Pahys J.M., Toll B.J., Kim A.J., Hwang S.W. (2022). Risk factors for proximal junctional kyphosis after pediatric spinal deformity surgery with halo gravity traction. Childs Nerv. Syst..

[2] Potaczek T., Jasiewicz B., Tesiorowski M., Zarzycki D., Szcześniak A. (2009). Treatment of idiopathic scoliosis exceeding 100 degrees-comparison of different surgical techniques. Ortop. Traumatol. Rehabil..

[3] Yilgor C., Kindan P., Yucekul A., Zulemyan T., Alanay A. (2022). Osteotomies for the Treatment of Adult Spinal Deformities: A Critical Analysis Review. JBJS Rev..

[4] Riley M.S., Lenke L.G., Chapman T.M., Sides B.A., Blanke K.M., Kelly M.P. (2018). Clinical and Radiographic Outcomes After Posterior Vertebral Column Resection for Severe Spinal Deformity with Five-Year Follow-up. J. Bone Jt. Surg..

[5] Koller H., Zenner J., Gajic V., Meier O., Ferraris L., Hitzl W. (2012). The impact of halo-gravity traction on curve rigidity and pulmonary function in the treatment of severe and rigid scoliosis and kyphoscoliosis: A clinical study and narrative review of the literature. Eur. Spine J..

[6] Liu D., Yang J., Sui W., Deng Y., Li F., Yang J., Huang Z. (2022). Efficacy of Halo-Gravity Traction in the Perioperative Treatment of Severe Scoliosis and Kyphosis: A Comparison of Adolescent and Adult Patients. World Neurosurg..

[7] Rocos B., Reda L., Lebel D.E., Dodds M.K., Zeller R. (2021). The Use of Halo Gravity Traction in Severe, Stiff Scoliosis. J. Pediatr. Orthop..

[8] LaMont L.E., Jo C., Molinari S., Tran D., Caine H., Brown K., Wittenbrook W., Schochet P., Johnston C.E., Ramo B. (2019). Radiographic, Pulmonary, and Clinical Outcomes with Halo Gravity Traction. Spinal Deform..

[9] Shi B., Liu D., Shi B., Li Y., Xia S., Jiang E., Qiu Y., Zhu Z. (2020). A Retrospective Study to Compare the Efficacy of Preoperative Halo-Gravity Traction and Postoperative Halo-Femoral Traction After Posterior Spinal Release in Corrective Surgery for Severe Kyphoscoliosis. Med. Sci. Monit..

[10] Rinella A., Lenke L., Whitaker C., Kim Y., Park S.S., Peelle M., Edwards C., Bridwell K. (2005). Perioperative halo-gravity traction in the treatment of severe scoliosis and kyphosis. Spine.

[11] Koller H., Mayer M., Koller J., Ferraris L., Wiedenhöfer B., Hitzl W., Hempfing A. (2021). Temporary treatment with magnetically controlled growing rod for surgical correction of severe adolescent idiopathic thoracic scoliosis greater than 100°. Eur. Spine J..

[12] Di Silvestre M., Zanirato A., Greggi T., Scarale A., Formica M., Vallerga D., Legrenzi S., Felli L. (2020). Severe adolescent idiopathic scoliosis: Posterior staged correction using a temporary magnetically controlled growing rod. Eur. Spine J..

[13] Lenke L.G. (2005). Lenke classification system of adolescent idiopathic scoliosis: Treatment recommendations. Instr. Course Lect..

[14] Skaggs D.L., Lee C., Myung K.S. (2014). Neuromonitoring Changes Are Common and Reversible with Temporary Internal Distraction for Severe Scoliosis. Spine Deform..

[15] Shimizu T., Lenke L.G., Cerpa M., Lehman R.A., Pongmanee S., Sielatycki J.A. (2020). Preoperative halo-gravity traction for treatment of severe adult kyphosis and scoliosis. Spine Deform..

[16] McIntosh A.L., Ramo B.S., Johnston C.E. (2019). Halo Gravity Traction for Severe Pediatric Spinal Deformity: A Clinical Concepts Review. Spine Deform..

[17] Pizones J., Sánchez-Mariscal F., Zúñiga L., Izquierdo E. (2015). Ponte osteotomies to treat major thoracic adolescent idiopathic scoliosis curves allow more effective corrective maneuvers. Eur. Spine J..

[18] Gottlich C., Sponseller P.D. (2020). Ponte Osteotomy in Pediatric Spine Surgery. JBJS Essent. Surg. Tech..

[19] Suk S.I., Lee C.K., Kim W.J., Chung Y.J., Park Y.B. (1995). Segmental pedicle screw fixation in the treatment of thoracic idiopathic scoliosis. Spine.

[20] Cheung J.P., Cahill P., Yaszay B., Akbarnia B.A., Cheung K.M. (2015). Special article: Update on the magnetically controlled growing rod: Tips and pitfalls. J. Orthop. Surg..

[21] Wang J., Han B., Hai Y., Su Q., Chen Y. (2021). How helpful is the halo-gravity traction in severe spinal deformity patients?: A systematic review and meta-analysis. Eur. Spine J..

[22] Mehrpour S., Sorbi R., Rezaei R., Mazda K. (2017). Posterior-only surgery with preoperative skeletal traction for management of severe scoliosis. Arch. Orthop. Trauma Surg..

[23] Zhang Y., Hai Y., Tao L., Yang J., Zhou L., Yin P., Pan A., Zhang Y., Liu C. (2019). Posterior Multiple-Level Asymmetrical Ponte Osteotomies for Rigid Adult Idiopathic Scoliosis. World Neurosurg..

[24] Floccari L.V., Poppino K., Greenhill D.A., Sucato D.J. (2021). Ponte osteotomies in a matched series of large AIS curves increase surgical risk without improving outcomes. Spine Deform..

[25] Zhou J., Wang R., Huo X., Xiong W., Kang L., Xue Y. (2020). Incidence of Surgical Site Infection After Spine Surgery: A Systematic Review and Meta-analysis. Spine.

[26] Watanabe K., Lenke L.G., Bridwell K.H., Kim Y.J., Hensley M., Koester L. (2010). Efficacy of perioperative halo-gravity traction for treatment of severe scoliosis (≥100°). J. Orthop. Sci..

[27] Deng H., Chan A.K., Ammanuel S., Chan A.Y., Oh T., Skrehot H.C., Edwards S., Kondapavulur S., Nichols A.D., Liu C. (2019). Risk factors for deep surgical site infection following thoracolumbar spinal surgery. J. Neurosurg. Spine.

